# METTL14‐Mediated m^6^A Methylation Preserves Cardiomyocyte Homeostasis and Attenuates Progeria‐Associated Cardiac Remodeling

**DOI:** 10.1111/acel.70765

**Published:** 2026-10-15

**Authors:** Shixiao Wang, Jie Zhang, Ying Ao, Dan Rao, Cong Yu, Chenzhong Xu, Ming Wang, Mingying Li, Xin Huang, Jianfei Zhang, Rui Mei, Zhen Zhang, Jie Sun, Juanli Duan, Ying Sun, Zimei Wang, Wen Su, Baohua Liu

**Affiliations:** ^1^ Shenzhen Key Laboratory for Systemic Aging and Intervention (SKL‐SAI), School of Basic Medical Sciences, Shenzhen University Shenzhen China; ^2^ National Health Commission Key Laboratory of Bone Aging Shenzhen University Medical School Shenzhen China; ^3^ Guangdong Key Laboratory for Biomedical Measurements and Ultrasound Imaging, National—Regional Key Technology Engineering Laboratory for Medical Ultrasound, School of Biomedical Engineering Shenzhen University Shenzhen China; ^4^ Shenzhen Key Laboratory of Cell Senescence and Regeneration Innovation, School of Basic Medical Sciences Shenzhen University Shenzhen China; ^5^ School of Basic Medical Sciences Wannan Medical University Wuhu China; ^6^ Department of Hepatobiliary Surgery Xi‐Jing Hospital, Fourth Military Medical University Xi'an China; ^7^ Jiangsu Key Laboratory of Geriatric Precision Medicine and Aging Intervention Xuzhou Medical University Xuzhou Jiangsu China

**Keywords:** cardiac aging, cardiac hypertrophy, HGPS, METTL14, METTL3

## Abstract

Hutchinson–Gilford progeria syndrome (HGPS) is a rare premature aging disorder most commonly caused by a de novo synonymous mutation (c.1824C>T) in the *LMNA* gene. This mutation activates aberrant splicing of prelamin A, leading to the production and accumulation of progerin, which drives accelerated aging, with patients predominantly succumbing to cardiovascular disease. However, while cardiac pathologies are well documented, the cell‐autonomous contribution of cardiomyocytes to HGPS‐associated cardiac remodeling remains poorly understood. Here, we generated a cardiomyocyte‐specific progerin‐expressing mouse model (*Myh6*‐Cre; *Lmna* ^fl/fl^) and found that selective progerin expression in cardiomyocytes was sufficient to recapitulate key cardiac features of HGPS, including myocardial fibrosis, cardiac hypertrophy, and progressive functional decline. Mechanistically, progerin‐expressing hearts exhibited a marked reduction in the N^6^‐methyladenosine (m^6^A) writer components METTL3/14, and knockdown of *Mettl3/14* in primary cardiomyocytes decreased the expression of calcium‐handling transcript (*Casq2*), while elevating senescence‐related (*Cdkn1a* and *Cdkn2a*) and pro‐inflammatory transcripts (*Ifna* and *Ifnb*). Importantly, cardiomyocyte‐targeted restoration of METTL14 partially ameliorated pathological cardiac remodeling and dysfunction in progeroid mice. Together, the data identify METTL14‐dependent m^6^A methylation as a critical regulator of cardiomyocyte integrity during progeria‐associated cardiac remodeling. Restoration of cardiac m^6^A writer activity may represent a potential therapeutic strategy for laminopathy‐related cardiovascular aging.

## Graphical abstract

Progerin accumulation downregulates the expression of m^6^A methyltransferases METTL3 and METTL14 in cardiac tissues, impairing m^6^A modification‐mediated regulation of target genes including *Casq2*, *Ifna* and *Ifnb*, leading to calcium signaling dysregulation and pro‐inflammatory activation in the aged heart.

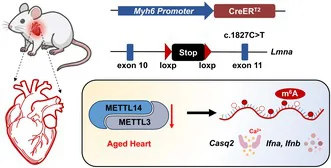

## 1 Background

Hutchinson–Gilford progeria syndrome (HGPS) is an extremely rare, fatal genetic disorder characterized by accelerated aging (DeBusk 1972; Merideth et al. 2008; Agarwal et al. 2010). The disease is predominantly caused by a point mutation (c.1824 C>T; p.Gly608=) in the *LMNA* gene, which activates a cryptic splice site and generates progerin—a toxic, permanently farnesylated mutant of Lamin A (Eriksson et al. 2003; Gordon et al. 2003). The accumulation of progerin disrupts the nuclear envelope architecture (Scaffidi and Misteli 2005) and triggers widespread cellular dysfunction (McCord et al. 2013; L. Wang et al. 2012; Sun et al. 2024), driving rapid systemic aging and a devastating clinical course characterized by a median life expectancy of only 14.6 years (Agarwal et al. 2010). Cardiovascular complications, including myocardial infarction and intractable heart failure, represent the primary cause of mortality (Merideth et al. 2008; Ullrich and Gordon 2015). While vascular stiffness is a well‐recognized systemic driver of cardiac load in HGPS (Prakash et al. 2018), the extent to which intrinsic cardiomyocyte defects drive the progressive heart failure remains a critical subject of debate (Chen et al. 2023).

As terminally differentiated cells, adult cardiomyocytes are not exempt from aging but are fundamentally predisposed to entering a senescent state marked by the accumulation of DNA damage (L. Wu et al. 2023), mitochondrial homeostatic dysregulation (Dai et al. 2010), and pathological hypertrophy (Anderson et al. 2019). Crucially, these senescent cardiomyocytes develop a senescence‐associated secretory phenotype (SASP), driving the robust release of diverse pro‐inflammatory cytokines and pro‐fibrotic factors (Lewis‐McDougall et al. 2019). This bioactive secretome acts via paracrine signaling to perpetuate a vicious cycle of chronic inflammation and maladaptive tissue remodeling within the local microenvironment (Childs et al. 2017). Ultimately, the accumulation of these senescent cells and their secretions exacerbate myocardial fibrosis and precipitate functional heart failure (Anderson et al. 2019).

N^6^‐methyladenosine (m^6^A) is the most prevalent internal modification in eukaryotic mRNA (Dominissini et al. 2012; Meyer et al. 2012), governed by a “writer” complex consisting of the catalytic core METTL3 and its structural partner METTL14 (Liu et al. 2014; Y. Wang et al. 2014). This m^6^A regulatory machinery is critical for mammalian organismal development, as its loss leads to severe developmental defects and embryonic lethality (Geula et al. 2015). Critically, dynamic fluctuations in the m^6^A landscape are emerging as a pivotal signature of organismal aging (Min et al. 2018). The deficiency of *Mettl3* or *Mettl14* is known to accelerate cellular senescence by destabilizing transcripts involved in DNA repair and stress responses (Z. Wu et al. 2020; Zhang et al. 2020). In the cardiovascular system, m^6^A homeostasis is equally indispensable (Mathiyalagan et al. 2019; Berulava et al. 2020). Recent studies have shown that m^6^A methyltransferases are required to prevent spontaneous cardiac remodeling; their absence leads to severe defects in sarcomere assembly and fatal dilated cardiomyopathy (Dorn et al. 2019; Kmietczyk et al. 2019). However, despite the established link between m^6^A loss and general cellular senescence, the epitranscriptomic landscape in the specific context of progeria remains uncharted.

In this study, using cardiomyocyte‐specific progeria mice, we identified significant downregulation of the methyltransferases METTL3 and METTL14 in the heart. This reduction negatively correlated with the expression of pro‐inflammatory factors and heart failure‐associated genes. Consistently, knockdown of *Mettl3*/*Mettl14* accelerated cardiomyocyte senescence and functional impairment. Conversely, cardiac‐specific restoration of METTL14 effectively attenuated cardiac fibrosis and extended the median survival of progeria mice. Our findings delineate a novel epitranscriptomic regulatory axis and identify a promising therapeutic target for HGPS‐associated cardiac complications.

## 2 Results

### 2.1 Cardiomyocyte‐Specific Expression of Progerin Induces Cardiac Aging Phenotypes Resembling HGPS

*LMNA*, encoding A‐type lamins, is a major disease gene in inherited cardiomyopathies, particularly dilated cardiomyopathy, underscoring the importance of nuclear lamina integrity in cardiomyocyte homeostasis (Supplementary Figure S1A). Given that progerin is a pathogenic lamin A variant that disrupts nuclear lamina architecture in HGPS, we hypothesized that its accumulation in cardiomyocytes might directly impair cardiac homeostasis and contribute to cardiomyopathy. To this end, we generated an inducible cardiomyocyte‐specific progerin‐expressing mouse model, *Myh6*‐Cre; *Lmna* ^fl/fl^, using a *Myh6*‐CreER^T2^/LoxP strategy in which progerin expression is induced by tamoxifen administration (Figure 1A,B). We first isolated primary cardiomyocytes from *Myh6*‐Cre; *Lmna* ^fl/fl^ mice and affirmed that 4‐Hydroxytamoxifen treatment triggered Cre‐mediated recombination at the *Lmna* locus, as shown by PCR detection of the progerin genomic sequence, whereas vehicle‐treated cells showed no comparable recombination signal (Figure S1B–D). Having validated Cre‐mediated recombination in isolated cardiomyocytes, we next examined the tissue specificity of progerin expression in vivo. Progerin transcripts were detected selectively in the hearts of tamoxifen‐treated *Myh6*‐Cre; *Lmna* ^fl/fl^ mice, but not in skeletal muscle or lung, with corn oil‐treated mice serving as vehicle controls (Figure 1C). Consistently, progerin protein was detected in cardiac lysates from tamoxifen‐treated mice while absent from non‐cardiac tissues and vehicle controls (Figure 1D). Immunofluorescence analysis further demonstrated that progerin accumulated at the nuclear envelope of cardiomyocytes, consistent with the expected subcellular distribution of mutant lamin A. Quantification of progerin‐positive signals revealed robust and sustained cardiac expression at both 1 and 8 months after tamoxifen induction in *Myh6*‐Cre; *Lmna* ^fl/fl^ mice (Figure 1E,F). Together, these data establish *Myh6*‐Cre; *Lmna* ^fl/fl^ mice as an inducible and cardiomyocyte‐restricted model for determining the cell‐autonomous role of progerin in HGPS‐associated cardiac aging and injury.

**FIGURE 1:**
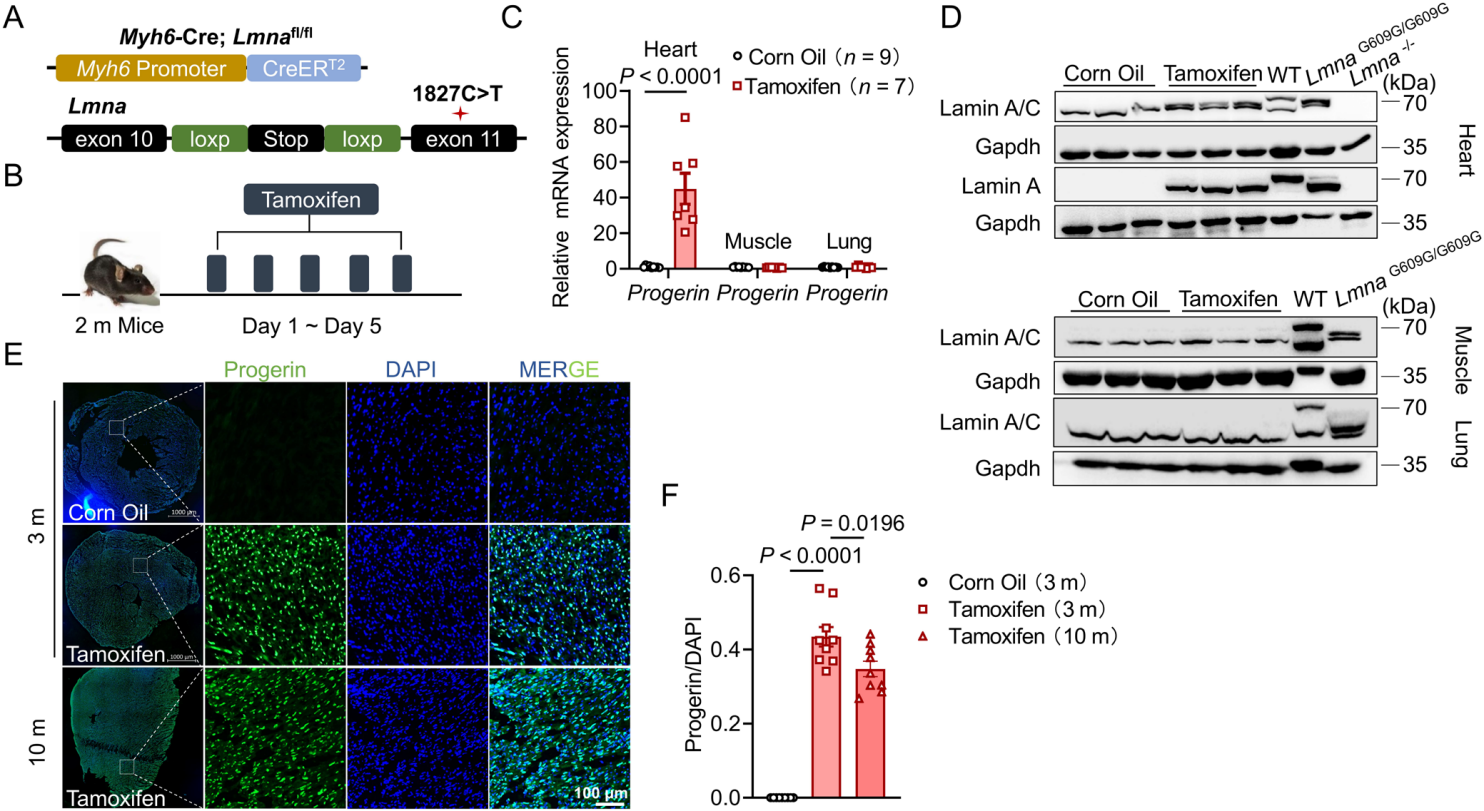
Generation and characterization of the cardiomyocyte‐specific progerin mouse model (*Myh6*‐Cre; *Lmna* ^fl/fl^). (A) Schematic representation of the gene targeting strategy. The *Myh6‐*CreER^T2^/LoxP system was employed to drive cardiac‐restricted expression of progerin by targeting intron 10 of the *Lmna* gene. (B) Experimental protocol for progerin induction. Two‐month‐old mice received intraperitoneal (i.p.) injections of tamoxifen for five consecutive days. Corn oil treatment served as the vehicle control. (C) qRT‐PCR quantification of progerin mRNA levels in various tissues of *Myh6*‐Cre; *Lmna* ^fl/fl^ mice post‐tamoxifen induction. Corn oil group: *N* = 9 biological replicates; Tamoxifen group: *N* = 7 biological replicates. (D) Representative western blots showing progerin protein levels across various tissues. (E) Representative immunofluorescence images showing the expression and subcellular localization of progerin (green) in cardiac sections. Nuclei were counterstained with DAPI (blue). Scale bar = 100 μm. (F) Quantification of progerin‐positive cell number normalized to DAPI‐positive cell number in heart tissues. Data are presented as mean ± s.e.m. Statistical significance was determined by unpaired Student's *t*‐test (*p* < 0.05).

We next defined the pathological consequences of sustained progerin accumulation in the hearts of *Myh6*‐Cre; *Lmna* ^fl/fl^ mice. Consistent with previous observations linking progerin to defective genome maintenance (Chen et al. 2023), cardiac sections from tamoxifen‐induced *Myh6*‐Cre; *Lmna* ^fl/fl^ mice showed a marked reduction in 53BP1 immunofluorescence signal compared with vehicle controls (Figure S2A), a pattern more consistent with altered DNA damage signaling than with a decrease in DNA damage per se. Masson's trichrome staining revealed increased collagen deposition in hearts from tamoxifen‐induced mice (Figure 2A), together with transcriptional upregulation of the fibrosis‐associated genes *Col1a1* and *Col3a1* (Figure 2B). In parallel, progerin‐expressing hearts developed an age‐dependent hypertrophic phenotype. Cardiomyocyte cross‐sectional area was not significantly altered at 5 months but was markedly increased at 10 months (Figure 2C). Notably, expression of the hypertrophic marker *Myh7* was already elevated at 5 months (Figure 2D), indicating that transcriptional remodeling precedes detectable structural hypertrophy. We further observed a broader stress‐response signature in tamoxifen‐induced *Myh6*‐Cre; *Lmna* ^fl/fl^ hearts, characterized by increased expression of the heart failure marker *Nppb*, as well as the pro‐inflammatory gene *Ifna* and *Ifnb*. These changes were accompanied by reduced expression of genes involved in calcium handling, including *Casq1*, *Casq2*, and *Stim1* (Figure 2E). Of note, hearts from *Lmna* ^G609G/G609G^ mice also exhibited repression of these calcium‐handling genes (*Casq1*, *Casq2*, *Stim1*; Figure S2B). The transcriptional downregulation of calcium‐handling genes has previously been reported in *Zmpste24* ^−/−^ mouse hearts, where their reduced expression coincides with perturbed calcium transients and arrhythmic phenotypes (Rivera‐Torres et al. 2016). Consistent with these molecular alterations, echocardiographic analysis revealed impaired cardiac performance, with reduced stroke volume and cardiac output, despite preserved ejection fraction (Figure 2F). Importantly, while parameters including heart weight‐to‐body weight ratio, treadmill endurance, rotarod performance, and cardiac tissue stiffness, as assessed by Young's modulus, were not significantly changed between 5‐month‐old groups, a markedly shortened median lifespan was observed in the tamoxifen‐treated *Myh6*‐Cre; *Lmna* ^fl/fl^ mice compared with vehicle controls (Figure S2C–G). Together, these findings suggest persistent progerin expression in cardiomyocytes is sufficient to induce progressive myocardial remodeling, functional decline, and premature mortality. Notably, the vehicle‐treated *Myh6*‐Cre; *Lmna* ^fl/fl^ mice showed a shorter median lifespan than that of wild‐type mice. Given the use of *Myh6*‐driven Cre system, we investigated whether the transgenic background influences endogenous cardiac gene expression. In support of this notion, *Myh6* mRNA level was significantly reduced in the myocardium of *Myh6*‐Cre; *Lmna* ^fl/fl^ mice compared with wild‐type controls (Figure S2H). It suggests that reduced endogenous *Myh6* expression may contribute to the baseline vulnerability of the *Myh6*‐Cre; *Lmna* ^fl/fl^ background, while tamoxifen‐induced progerin expression further accelerates cardiac deterioration.

**FIGURE 2:**
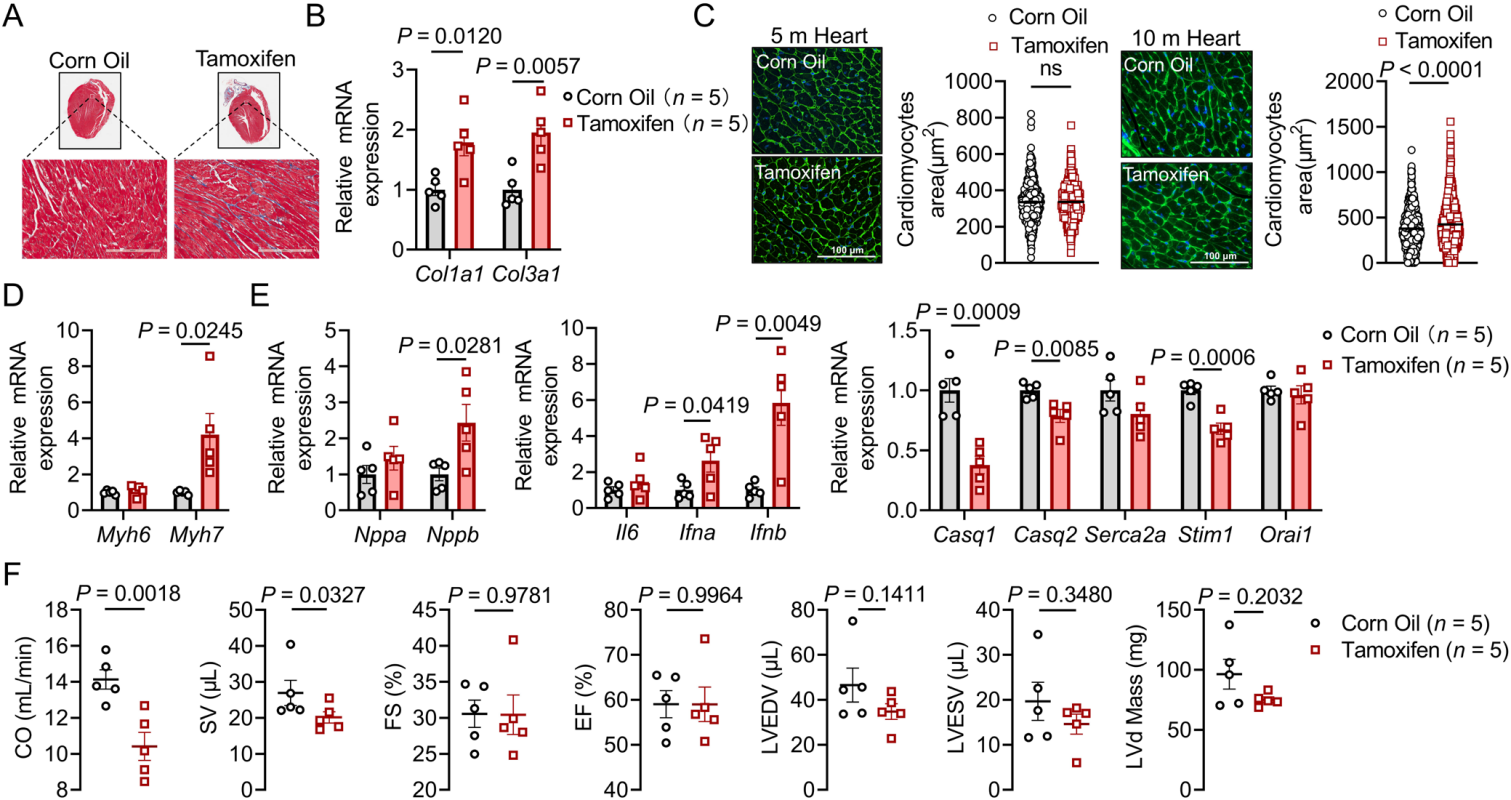
HGPS‐related cardiac pathological phenotypes in *Myh6*‐Cre; *Lmna* ^fl/fl^ mice. (A) Representative Masson's trichrome staining of cardiac sections from 5‐month‐old *Myh6*‐Cre; *Lmna* ^fl/fl^ mice. Collagen fibers (indicating fibrosis) are stained blue, and cardiomyocytes are stained red. Scale bar = 200 μm. (B) qRT‐PCR quantification of fibrosis‐associated genes (*Col1a1* and *Col3a1*) in the cardiac tissues of *Myh6*‐Cre; *Lmna* ^fl/fl^ mice (*n* = 5 biological replicates per group). (C) Representative Wheat Germ Agglutinin (WGA, green) staining and quantification of cardiomyocyte cross‐sectional area in 5‐ and 10‐month‐old *Myh6*‐Cre; *Lmna* ^fl/fl^ mice (*n* = 5 biological replicates per group, with > 1000 transversely sectioned cardiomyocytes analyzed per group). Nuclei were counterstained with DAPI (blue). Scale bar = 100 μm. (D, E) qRT‐PCR quantification of mRNA levels for genes related to hypertrophy (*Myh6, Myh7*) (D), heart failure (*Nppa*, *Nppb*), inflammation (*Il6*, *Ifna*, *Ifnb*), and calcium handling (*Casq1*, *Casq2*, *Serca2a*, *Stim1*, *Orai1*) (E) in cardiac tissues from 5‐month‐old *Myh6*‐Cre; *Lmna* ^fl/fl^ mice treated with corn oil or tamoxifen (*n* = 5 biological replicates per group). (F) Echocardiographic assessment of cardiac function in 5‐month‐old *Myh6*‐Cre; *Lmna* ^fl/fl^ mice, including Stroke Volume (SV), Cardiac Output (CO), Ejection Fraction (EF), Fractional Shortening (FS), Left Ventricular End‐Diastolic Volume (LVEDV), Left Ventricular End‐Systolic Volume (LVESV), and Left Ventricular (LV) Mass (*n* = 5 biological replicates per group). Data are presented as mean ± s.e.m. Statistical significance was determined by unpaired Student's *t*‐test (*p* < 0.05).

### 2.2 Downregulation of METTL3/14 in Progeroid and Aged Mouse Hearts

Given the essential role of m^6^A RNA methylation in cardiac homeostasis, we examined whether the m^6^A writer components METTL3 and METTL14 are involved in progerin‐driven cardiac injury. Indeed, a marked reduction of METTL3 and METTL14 proteins was revealed in *Myh6*‐Cre; *Lmna* ^fl/fl^ hearts, as determined by immunoblot analysis (Figure 3A). At the transcript level, *Mettl14* expression was significantly decreased in progerin‐expressing hearts, whereas *Mettl3* mRNA abundance remained largely unchanged (Figure 3B). To determine whether this defect was confined to the *Myh6*‐Cre; *Lmna* ^fl/fl^ model or represented a broader feature of progeroid cardiac stress, we examined two systemic progeria models: *Lmna* ^G609G/G609G^ mice, which accumulate progerin, and *Zmpste24* ^−/−^ mice, which accumulate prelamin A. Consistently, both models exhibited significant downregulation of METTL3 and METTL14 protein levels in the heart tissues (Figure 3C–E). In line with the downregulation of METTL3 and METTL14 proteins, global m^6^A RNA methylation abundance was markedly decreased in tamoxifen‐induced *Myh6*‐Cre; *Lmna* ^fl/fl^ and *Lmna* ^G609G/G609G^ heart tissues, as assessed by m^6^A dot‐blot assay (Figure 3F,G). Given that progeroid syndromes recapitulate key aspects of aging, we further examined whether loss of m^6^A writers also occurs during physiological cardiac aging. Hearts from naturally aged wild‐type mice showed significantly reduced METTL3 and METTL14 abundance compared with young controls (Figure 3H and Figure S3A,B). A similar decrease in METTL14 was also observed in the hearts of *Lmna* knockout mice (Figure S3D), suggesting that distinct forms of lamin perturbation converge on attenuation of the cardiac m^6^A writer machinery. Interestingly, the protein levels of METTL3 or METTL14 were not altered in kidney tissues from naturally aged mice or *Lmna* knockout mice (Figure S3C,E), indicating that this response is tissue specific. In addition, ectopic expression of lamin A in 293 T cells increased METTL14 protein abundance (Figure S3F), supporting a molecular link between lamin A and the m^6^A writer complex. Together, these data identify suppression of METTL3 and METTL14 as a recurrent molecular feature of progeroid and physiologically aged hearts, and position loss of m^6^A writer activity as a cardiac‐selective epitranscriptomic signature of lamin‐associated aging.

**FIGURE 3:**
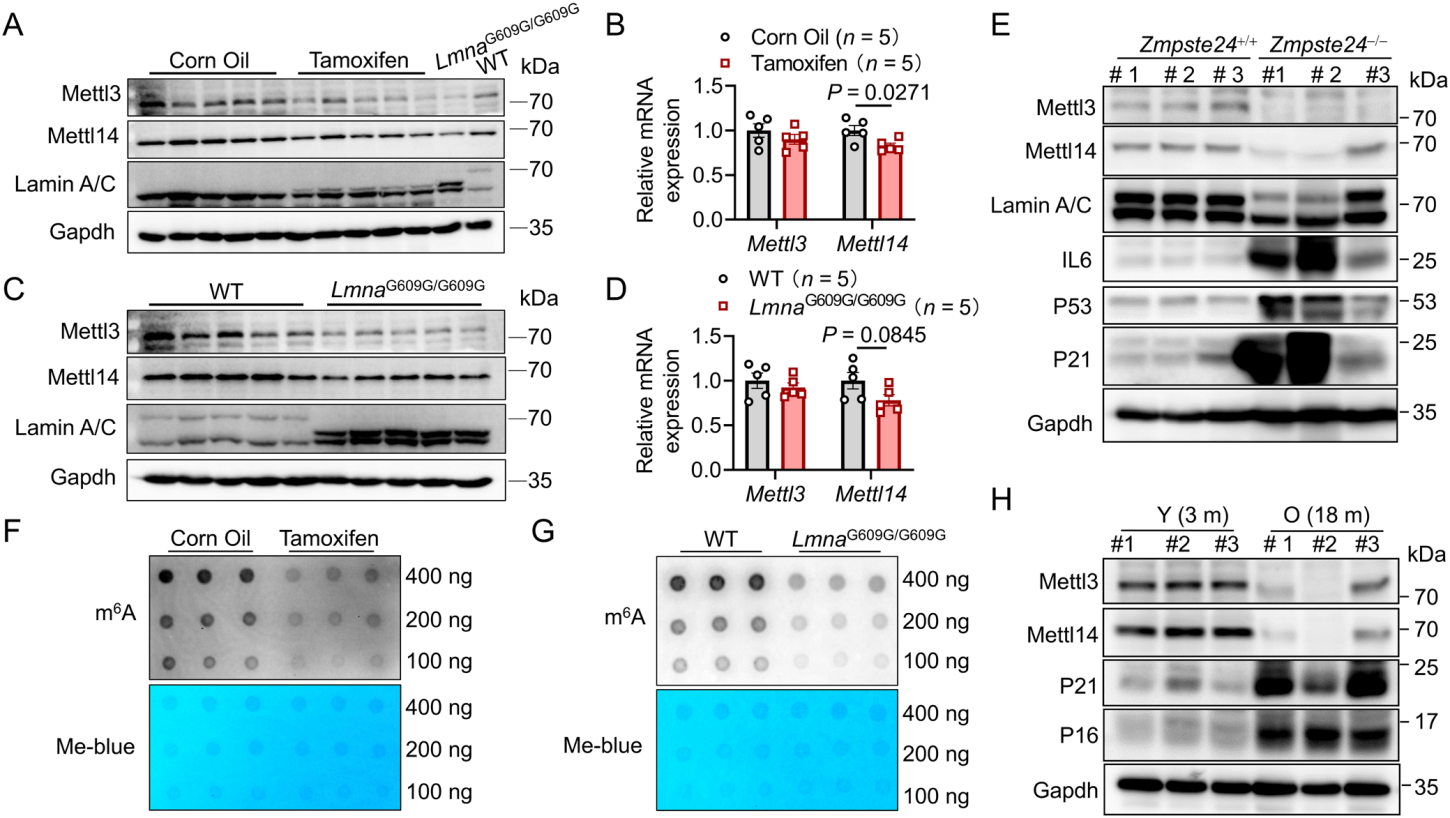
METTL3 and METTL14 expression in progeroid and naturally aged mouse heart. (A) Western blot analysis of METTL3 and METTL14 in cardiac tissues of 5‐month‐old *Myh6*‐Cre; *Lmna* ^fl/fl^ mice. (B) qRT‐PCR analysis of *Mettl3* and *Mettl14* in cardiac tissues of 5‐month‐old *Myh6*‐Cre; *Lmna* ^fl/fl^ mice (*n* = 5 biological replicates per group). (C) Western blot analysis of METTL3 and METTL14 in cardiac tissues of 4‐month‐old *Lmna* ^G609G/G609G^ progeria and wild‐type (WT) mice. (D) qRT‐PCR analysis of *Mettl3* and *Mettl14* in cardiac tissues of 4‐month‐old *Lmna* ^G609G/G609G^ progeria and wild‐type (WT) mice (*n* = 5 biological replicates per group). (E) Western blot analysis of METTL3 and METTL14 in cardiac tissues of 4‐month‐old *Zmpste24* ^*−/−*^ progeria and WT mice. (F, G) m^6^A dot‐blot assay detecting global m^6^A abundance in total RNA extracted from cardiac tissues of 5‐month‐old *Myh6*‐Cre; *Lmna* ^fl/fl^ mice treated with tamoxifen (with corn oil as control) (F) and 4‐month‐old *Lmna* ^G609G/G609G^ mice (with WT mice as control) (G). (H) Western blot analysis of METTL3 and METTL14 in cardiac tissues of young (3‐month‐old) and naturally aged (18‐month‐old) wild‐type mice. Data are presented as mean ± s.e.m. Statistical significance was determined by unpaired Student's *t*‐test (*p* < 0.05).

### 2.3 Depletion of *Mettl3/14* Causes Cardiomyocyte Senescence and Calcium‐Handling Defects

We next sought to determine whether this epitranscriptomic decline is causally linked to cardiomyocyte dysfunction. To this end, we silenced *Mettl3* and *Mettl14* individually or in combination in primary neonatal cardiomyocytes. Efficient depletion of METTL3 and METTL14 was confirmed at both protein and mRNA levels (Figure 4A,B). Notably, silencing of METTL3/14 led to a concomitant upregulation of the senescence markers p21 and p16 (Figure 4A). Notably, knockdown of either *Mettl3* or *Mettl14* induced the expression of pro‐inflammatory genes, including *Ifna*, *Ifnb* and *Il6*, as well as *Cdkn1a* and *Cdkn2a*, with combined depletion producing the strongest induction (Figure 4C). To determine whether these pro‐inflammatory transcripts undergo m^6^A modification in vivo, MeRIP‐qPCR was performed. We found that cardiac tissues from *Lmna* ^G609G/G609G^ mice exhibited reduced m^6^A enrichment within *Ifna* and *Ifnb* transcripts, compared to wild‐type controls (Figure 4D). Furthermore, RNA decay assays in actinomycin‐D‐treated cardiomyocytes demonstrated that silencing of *Mettl14* significantly prolonged the half‐lives of *Ifna* and *Ifnb* mRNAs (Figure 4E), indicating that m^6^A modification promotes their degradation. Moreover, METTL3 and METTL14 depletion recapitulated calcium‐handling defects. Knockdown of *Mettl3*, *Mettl14*, or both significantly suppressed key calcium‐handling genes, particularly *Casq1* and *Casq2* (Figure 4F). Consistently, MeRIP and RIP‐qPCR analyses confirmed a marked reduction in m^6^A levels and METTL14 binding on *Casq1* and *Casq2* mRNAs in *Lmna* ^G609G/G609G^ hearts (Figure 4G). Contrasting to pro‐inflammatory genes, silencing of *Mettl14* accelerated the decay of *Casq2* mRNA, while the stability of *Casq1* mRNA remained virtually unaffected (Figure 4H). Given the critical role of *Casq1* and *Casq2* in calcium signaling, we evaluated the physiological impact of *Mettl14* deficiency on cardiomyocytes. Intracellular calcium imaging showed that *Mettl14* silencing markedly reduced the peak amplitude of spontaneous calcium transients (Δ*F*/*F*_*0*_) in primary cardiomyocytes (Figure 4I), consistent with altered calcium handling. To dissect whether the calcium‐handling targets *Casq1* and *Casq2* directly drive cardiac aging‐associated pathological phenotypes, we individually knocked down *Casq1* or *Casq2* in cardiomyocytes. Interestingly, knockdown of *Casq2* significantly upregulated the expression of the fibrotic gene *Col3a1* and senescence markers *Cdkn1a* and *Cdkn2a*, as determined by qRT‐PCR, whereas *Casq1* knockdown had little effect (Figure 4J). Collectively, these findings support a model in which progerin affects the m^6^A modification of transcripts involved in inflammation and calcium handling, thereby leading to cardiomyocyte dysfunction and loss of homeostasis.

**FIGURE 4:**
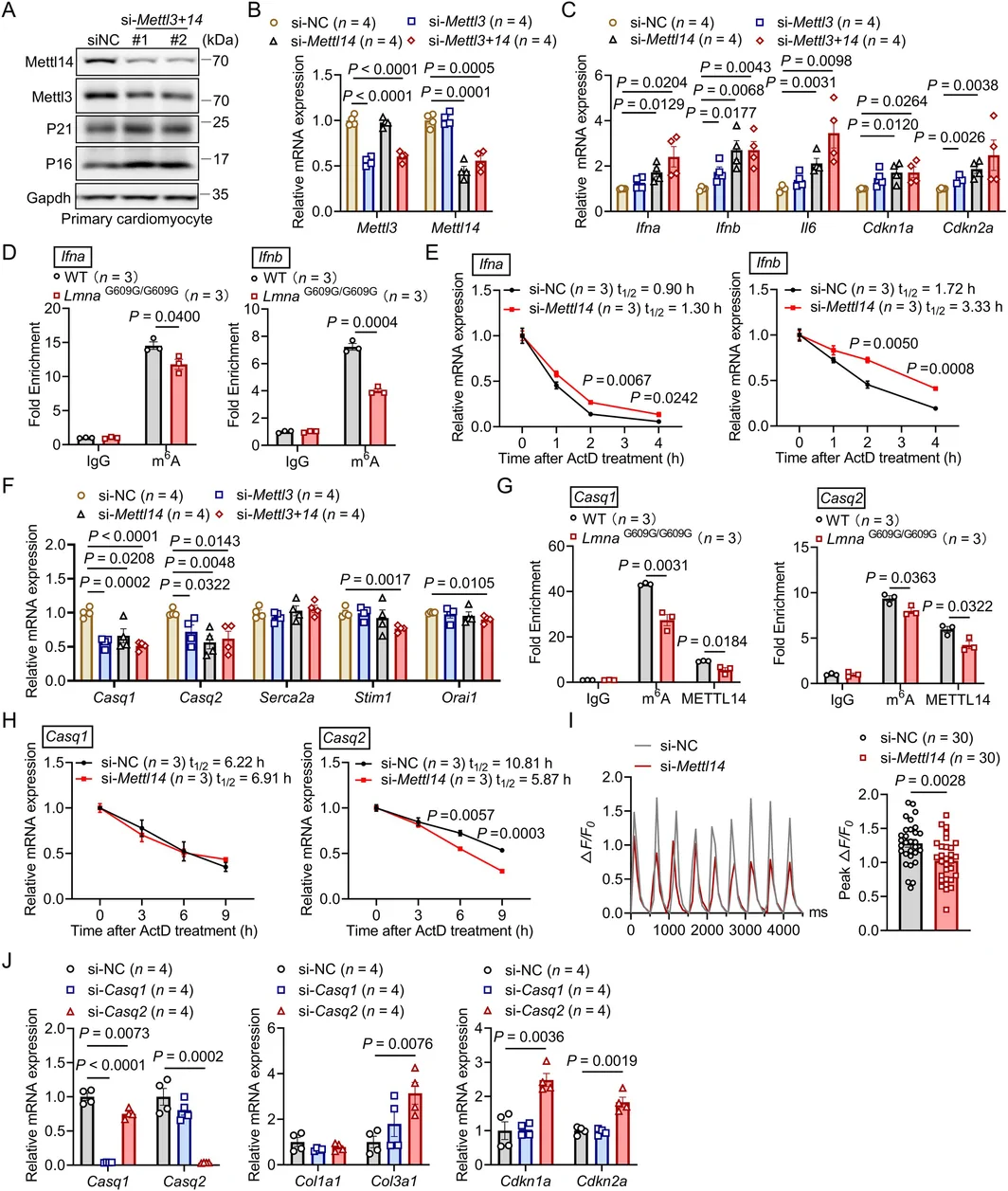
*Mettl3/14* deficiency dysregulates m^6^A‐regulated pro‐inflammatory and calcium‐handling transcripts and promotes cardiomyocyte senescence. (A) Western blot analysis of METTL3/METTL14 and senescence markers (p16 and p21) in primary cardiomyocytes following siRNA‐mediated knockdown of *Mettl3* and *Mettl14*. (B) qRT‐PCR quantification of *Mettl3*/*14* mRNA levels in primary cardiomyocytes transfected with si‐NC, si*‐Mettl3*, si*‐Mettl14*, or both (*n* = 4 replicates per group). (C) qRT‐PCR quantification of inflammation‐ and senescence‐associated mRNA levels in primary cardiomyocytes transfected with si‐NC, si‐*Mettl3*, si‐*Mettl14*, or both (*n* = 4 replicates per group). (D) MeRIP assays showing m^6^A enrichment on *Ifna* and *Ifnb* mRNAs in heart tissues from WT and *Lmna* ^G609G/G609G^ mice. IgG was used as a negative control (*n* = 3 replicates per group). (E) Actinomycin‐D‐chase mRNA stability assays measuring the transcript decay of *Ifna* and *Ifnb* in primary cardiomyocytes transfected with si‐NC or si‐*Mettl14* (*n* = 3 biological replicates per group). (F) qRT‐PCR quantification of mRNA levels for genes related to calcium‐handling (*Casq1*, *Casq2, Serca2a, Stim1*, and *Orai1*) in primary cardiomyocytes transfected with si‐NC, si*‐Mettl3*, si*‐Mettl14*, or both (*n* = 4 replicates per group). (G) MeRIP and METTL14 RIP‐qPCR assays showing m^6^A enrichment and METTL14‐RNA binding on *Casq1* and *Casq2* mRNAs in heart tissues from *Lmna* ^G609G/G609G^ and WT mice. IgG was used as a negative control (*n* = 3 replicates per group). (H) Actinomycin‐D‐chase mRNA stability assays measuring the transcript decay of *Casq1* and *Casq2* in primary cardiomyocytes transfected with si‐NC or si‐*Mettl14* (*n* = 3 biological replicates per group). (I) Calcium transient measurements in primary cardiomyocytes transfected with si‐NC or si‐*Mettl14*. Representative traces of spontaneous calcium transients (Δ*F/F*_*0*_) are shown on the left. Quantitative analysis of peak Δ*F/F*_*0*_ amplitude is presented on the right, *n* = 30 cells from 3 independent cardiomyocyte isolations (biological replicates) per group. (J) qRT‐PCR quantification of mRNA levels for genes related to fibrosis (*Col1a1* and *Col3a1*) and senescence (*Cdkn1a* and *Cdkn2a*) in primary cardiomyocytes transfected with si‐NC, si‐*Casq1*, or si‐*Casq2* (*n* = 4 biological replicates per group). Data are presented as mean ± s.e.m. Statistical significance was determined by unpaired Student's *t*‐test (*p* < 0.05).

### 2.4 Restoration of METTL14 Mitigates Progeria‐Associated Cardiac Aging

Having established that METTL14 deficiency promotes cardiomyocyte senescence and disrupts calcium handling, we sought to determine whether restoring METTL14 could confer cardioprotection. We first observed that METTL14 reduction is not restricted to laminopathy, as isoproterenol (ISO) treatment markedly reduced its protein abundance in primary cardiomyocytes (Figure S4A,B), indicating that METTL14 loss is a common feature of cardiomyocyte stress. Based on these findings, we investigated whether targeted METTL14 replenishment could mitigate progeria‐associated cardiac pathology in vivo. To this end, we delivered METTL14 into 2‐month‐old *Myh6*‐Cre; *Lmna* ^fl/fl^ mice using an AAV9 vector driven by the cardiac troponin T (cTnT) promoter, with a GFP reporter to monitor transduction efficiency and myocardial tropism (Figure S4C). Phenotypic analyses were performed 2 months after AAV injection. Immunoblotting confirmed successful METTL14 overexpression in *Myh6*‐Cre; *Lmna* ^fl/fl^ hearts, with a concomitant increase in endogenous METTL3 level (Figure 5A). At the same time, this replenishment restored the expression of calcium‐handling genes (*Casq1* and *Casq2*) and suppressed pro‐inflammatory genes (*Ifna* and *Ifnb*) (Figure 5B). In addition, METTL14 overexpression reduced the expression of fibrosis‐associated genes *Col1a1* and *Col3a1* (Figure 5C). Consistent with these molecular changes, Masson's trichrome staining showed reduced myocardial collagen deposition and alleviated fibrotic remodeling following METTL14 restoration (Figure 5D). Given these protective effects in the *Myh6*‐Cre; *Lmna* ^fl/fl^ cardiomyocyte‐specific progeria model, we next investigated whether METTL14 replenishment could similarly rescue cardiac phenotypes in mice with systemic premature aging. Two‐month‐old *Zmpste24* ^−/−^ mice received tail‐vein injection of AAV9 vectors, either driven by the ubiquitous CMV promoter or by the cTnT promoter, and phenotypic analyses were performed 2 months after AAV injection. GFP fluorescence confirmed efficient viral delivery, with widespread expression after AAV‐CMV‐METTL14‐GFP administration and myocardium‐restricted expression after AAV‐cTnT‐METTL14‐GFP administration (Figure 5E). Consistent with these imaging results, qRT‐PCR analysis verified elevated *Mettl14* transcript levels in hearts receiving AAV‐cTnT‐METTL14‐GFP (Figure 5F). Following cardiomyocyte‐specific METTL14 overexpression, we further found that it restored the expression of the calcium‐handling gene *Casq2*, while suppressing genes associated with inflammation (*Ifnb*), fibrosis (*Col1a1* and *Col3a1*), and heart failure (*Nppa* and *Nppb*) (Figure 5G–J). Interestingly, senescence‐associated markers (*Cdkn1a* and *Cdkn2a*) remained unchanged (Figure S4D). In addition, AAV‐cTnT‐METTL14‐GFP treatment markedly reduced myocardial collagen deposition in hearts from *Zmpste24* ^−/−^ mice, as evidenced by Masson's trichrome staining (Figure 5K). These cardiac improvements translated into enhanced organismal performance, evidenced by increased latency to fall in the rotarod assay (Figure 5L) and extended median lifespan of *Zmpste24* ^−/−^ mice compared with control‐treated littermates (Figure 5M). Collectively, METTL14 restoration produced consistent protective effects in both cardiomyocyte‐specific and systemic models of premature aging, including improved calcium‐handling and pro‐inflammatory gene expression, attenuated pathological fibrotic remodeling, and improved overall disease outcomes. Therefore, maintenance of cardiac m^6^A writer axis integrity may represent a therapeutically modifiable factor in progeria‐associated cardiac aging and disease progression.

**FIGURE 5:**
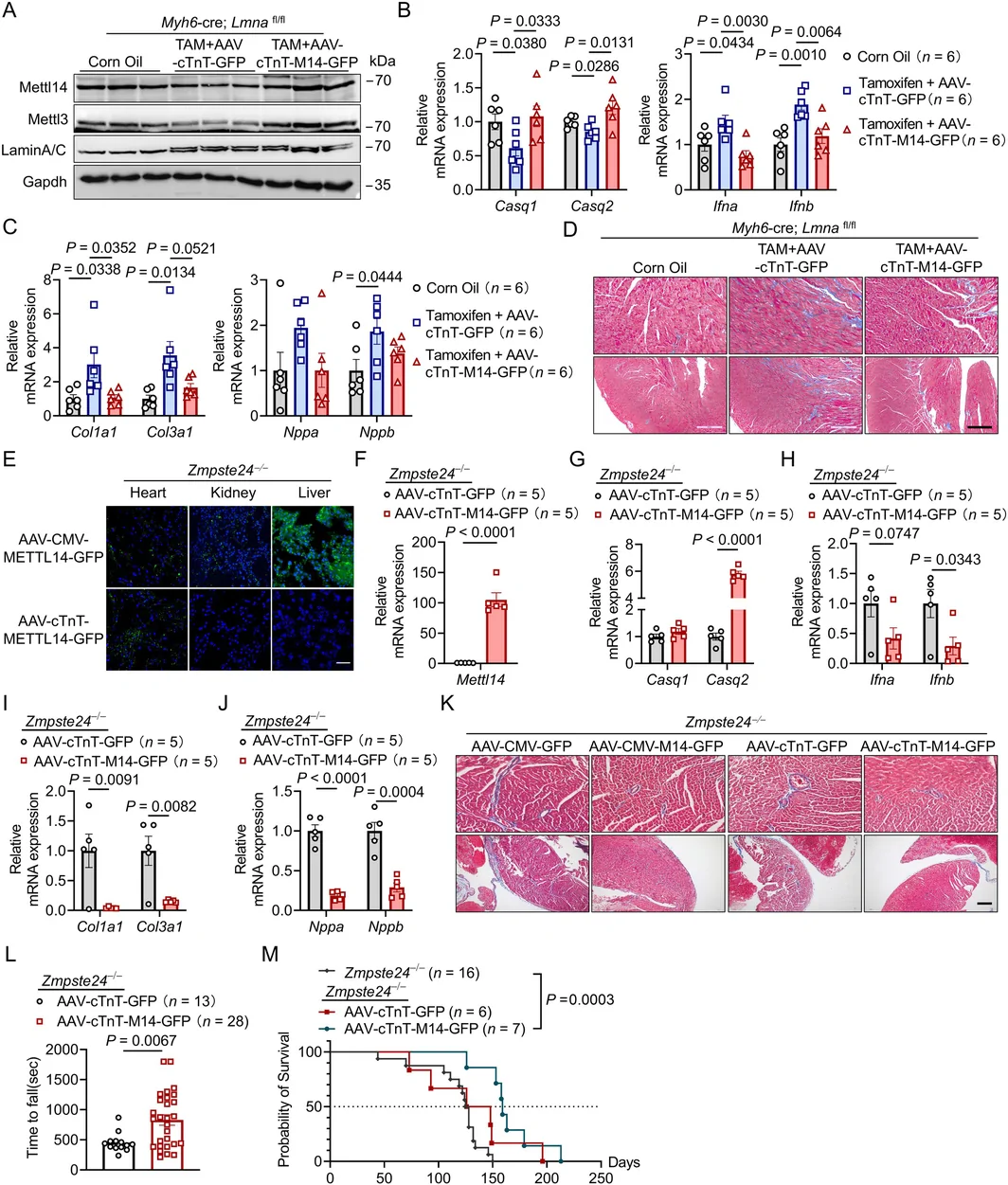
Cardiomyocyte‐specific restoration of METTL14 ameliorates cardiac pathology in *Myh6*‐Cre; *Lmna* ^fl/fl^ and *Zmpste24* ^−/−^ progeroid mouse models. (A) Western blot analysis of METTL14 and METTL3 protein levels in hearts from *Myh6*‐Cre; *Lmna* ^fl/fl^ mice treated with AAV‐cTnT‐GFP and AAV‐cTnT‐METTL14‐GFP. (B, C) qRT‐PCR quantification of mRNA levels for genes related to calcium‐handling (*Casq1* and *Casq2*), inflammation (*Ifna* and *Ifnb*) (B), fibrosis (*Col1a1* and *Col3a1*), and heart failure (*Nppa* and *Nppb*) (C) in heart tissues of *Myh6*‐Cre; *Lmna* ^fl/fl^ mice treated with tamoxifen in combination with AAV‐cTnT‐GFP or AAV‐cTnT‐METTL14‐GFP, with corn oil as the vehicle control (*n* = 6 biological replicates per group). (D) Representative images of Masson's trichrome staining in cardiac tissues of *Myh6*‐Cre; *Lmna* ^fl/fl^ mice treated with AAV‐cTnT‐GFP and AAV‐cTnT‐METTL14‐GFP. Scale bar = 500 μm. (E) Representative immunofluorescence images validating adeno‐associated virus (AAV9) transduction. GFP signals (green) demonstrate systemic expression in the AAV‐CMV‐METTL14‐GFP group, whereas expression is restricted to the myocardium in the AAV‐cTnT‐METTL14‐GFP group. Scale bar = 100 μm. (F) qRT‐PCR analysis of *Mettl14* in cardiac tissues of *Zmpste24* ^−/−^ mice treated with AAV‐cTnT‐GFP or AAV‐cTnT‐METTL14‐GFP (*n* = 5 biological replicates per group). (G–J) qRT‐PCR quantification of mRNA levels for genes related to calcium‐handling (*Casq1* and *Casq2*) (G), inflammation (*Ifna* and *Ifnb*) (H), fibrosis (*Col1a1* and *Col3a1*) (I), and heart failure (*Nppa* and *Nppb*) (J) in heart tissues of *Zmpste24* ^−/−^ mice treated with AAV‐cTnT‐GFP or AAV‐cTnT‐METTL14‐GFP (*n* = 5 biological replicates per group). (K) Representative images of Masson's trichrome staining in cardiac tissues of *Zmpste24* ^−/−^ mice treated with AAV‐cTnT‐GFP and AAV‐cTnT‐METTL14‐GFP. Scale bar = 100 μm. (L) Motor coordination and endurance assessed by the rotarod test. Comparison of latency to fall between *Zmpste24* ^−/−^ mice (*n* = 13) and the METTL14‐supplemented *Zmpste24* ^−/−^group (*n* = 28). (M) Kaplan–Meier survival analysis of *Zmpste24* ^−/−^ mice. Restoration of cardiac METTL14 (*n* = 7) significantly extended the lifespan compared to the control *Zmpste24* ^−/−^ group (*n* = 16). Statistical significance was determined by the log‐rank (Mantel‐Cox) test (*p* < 0.05). Data are presented as mean ± s.e.m. Statistical significance was determined by unpaired Student's *t*‐test (*p* < 0.05).

## 3 Discussion

In the present study, by establishing the *Myh6*‐Cre; *Lmna* ^fl/fl^ mouse model, we provide evidence that cardiomyocyte‐specific nuclear envelope dysfunction is sufficient to trigger progressive fibrosis and functional decline. Our findings identify METTL3/14 downregulation as an important contributor to cardiomyocyte senescence, linking RNA methylation loss to progeria. Global and transcript‐specific m^6^A methylation is reduced in progeroid hearts, which impairs calcium handling, while cardiomyocyte‐targeted METTL14 restoration ameliorates cardiac pathology. This work not only broadens our understanding of progeria‐associated cardiovascular decline but also positions m^6^A regulation as an important layer of epitranscriptomic control in cardiac aging.

Although METTL3 and METTL14 were both downregulated in progeroid hearts, we focused our functional investigations primarily on METTL14. This decision was made firstly because our previous finding revealed a stronger physical interaction between lamin A and METTL14 compared with METTL3, suggesting a more direct role for METTL14 in nuclear envelope integrity (Zhang et al. 2020). Secondly, this decision was supported by the mutual stabilization of METTL3 and METTL14 (Y. Wang et al. 2014), and our in vivo observation that METTL14 overexpression elevated endogenous METTL3 protein levels in *Myh6*‐Cre; *Lmna* ^fl/fl^ mice. These data provide evidence that exogenous METTL14 is capable of stabilizing residual endogenous METTL3, thereby reconstituting a functional m^6^A writer complex without necessity of co‐overexpression of METTL3. This provides a strong rationale for METTL14 monotherapy and guides future comparisons of METTL3‐only or combined METTL3/METTL14 rescue.

Beyond m^6^A writer complex assembly, METTL14‐dependent mRNA methylation is vital for cardiomyocyte homeostasis. Our investigation revealed that METTL14 was associated with key calcium‐handling (*Casq2*) and pro‐inflammatory (*Ifna* and *Ifnb*) transcripts. Mechanistically, m^6^A modification differentially regulates these transcripts: reduced m^6^A on *Ifna* and *Ifnb* increases their mRNA stability and drives inflammatory activation, whereas reduced m^6^A on *Casq2* accelerates its decay and impairs calcium handling. Notably, although m^6^A modification was detected on both *Casq1* and *Casq2* transcripts, *Mettl14* silencing altered only the mRNA stability of *Casq2*, whereas the mRNA half‐life of *Casq1* remained largely unchanged. Nevertheless, total *Casq1* mRNA abundance was still reduced upon *Mettl14* knockdown, suggesting that *Casq1* downregulation occurs through additional layers of regulation. Notably, although both *Casq1* and *Casq2* are downregulated under progerin‐mediated stress, only knockdown of *Casq2*, but not *Casq1*, induces pro‐senescent and pro‐fibrotic transcriptional responses in cardiomyocytes. This phenotypic disparity may be attributed to previously reported expression profiles indicating that *Casq2* is the predominant calsequestrin isoform in the heart, whereas *Casq1* exhibits extremely low basal expression in cardiac tissue (Rossi et al. 2021). These findings suggest that *Casq2*, rather than *Casq1*, acts as the key regulatory isoform driving the progression of cardiac aging and dysfunction. In addition, although our data implicate calcium‐handling and pro‐inflammatory transcripts as functionally relevant METTL14‐associated targets, METTL14 likely regulates a broader RNA network involved in cardiac stress responses, inflammation and cellular senescence. Future transcriptome‐wide m^6^A profiling and METTL14‐RNA interactome analyses will be important to define the full repertoire of METTL14‐dependent targets in progeroid myocardium. Meanwhile, future studies should determine whether METTL14 restoration can reverse established cardiac pathology or primarily acts by preventing disease progression.

An important finding in our study is that cardiomyocyte‐targeted METTL14 restoration produced consistent protective effects in two independent progeria models. In *Myh6*‐Cre; *Lmna* ^fl/fl^ mice, METTL14 overexpression restored *Casq1* and *Casq2* expression, suppressed *Ifna*/*Ifnb* and *Col1a1*/*Col3a1*, and reduced cardiac fibrosis. In systemic *Zmpste24* ^−/−^ mice, METTL14 restoration increased *Casq2*, suppressed *Ifnb*, *Col1a1*, *Col3a1*, *Nppa*, and *Nppb*, reduced myocardial collagen deposition, improved rotarod performance, and significantly extended median lifespan. These results demonstrate that correcting cardiac m^6^A writer activity can provide substantial survival benefit, even in a systemic progeroid model. Nonetheless, our current analysis of cardiac phenotypes is limited to baseline structural and functional readouts. Future studies employing echocardiography and ECG telemetry will further delineate the functional and rhythm consequences of cardiomyocyte progerin accumulation in progeria‐associated cardiac remodeling.

In addition, the genetic background of the *Myh6*‐Cre; *Lmna* ^fl/fl^ model should be considered when interpreting the survival phenotype. Given that *Myh6* deficiency is known to impair cardiac function and survival (Jones et al. 1996), the background‐associated suppression of *Myh6* in *Myh6*‐Cre; *Lmna* ^fl/fl^ mice may contribute to their reduced lifespan. In this littermate‐controlled comparison, progerin induction further accelerated cardiac deterioration and shortened lifespan, supporting a cardiomyocyte‐intrinsic effect. In conclusion, our results provide a rationale for exploring METTL14 as a potential therapeutic target for HGPS‐associated cardiovascular complications.

## 4 Materials and Methods

### 4.1 Mice

SPF‐grade *Myh6*‐Cre; *Lmna* ^fl/fl^ mice and *Zmpste24* ^−/−^ progeria mice were purchased from Cyagen Biosciences. All mice were housed in an SPF‐grade animal facility with a temperature of 22°C ± 2°C, humidity of 50% ± 5%, and a 12 h light/dark cycle, with free access to food and water. All animal experiments were approved by the Institutional Animal Care and Use Committee of Shenzhen University (Approval No. 202300033).

#### 4.1.1 Tamoxifen Induction of *Myh6*‐Cre; *Lmna* ^fl/fl^ Mice

Two‐month‐old mice were randomly assigned to receive intraperitoneal (i.p.) injections of either corn oil (Aladdin, 8001‐30‐7; 10 μL/g) or tamoxifen (Sigma‐Aldrich, T5648; 10 mg/mL; 10 μL/g) for 5 consecutive days.

#### 4.1.2 AAV‐Mediated METTL14 Restoration in *Myh6*‐Cre; *Lmna* ^fl/fl^ Mice

Two‐month‐old mice were injected via the tail vein with either AAV‐cTnT‐GFP (1 × 10^12^ vg/mL, 100 μL per mouse; Hanbio Biotechnology) or AAV‐cTnT‐METTL14‐GFP (1 × 10^12^ vg/mL, 100 μL per mouse; Hanbio Biotechnology). Phenotypic analyses were performed at 2 months post‐injection.

#### 4.1.3 AAV‐Mediated METTL14 Restoration in *Zmpste24* ^−/−^ Progeria Mice

Two‐month‐old *Zmpste24* ^−/−^ mice received tail‐vein injections of AAV vectors (1 × 10^12^ vg/mL, 100 μL per mouse; Hanbio Biotechnology) divided into four groups: AAV‐CMV‐GFP, AAV‐cTnT‐GFP, AAV‐CMV‐METTL14‐GFP and AAV‐cTnT‐METTL14‐GFP. Phenotypic analyses were performed at 2 months post‐injection.

### 4.2 Isolation and Transfection of Primary Cardiomyocytes

#### 4.2.1 Isolation and Culture of Primary Neonatal Mouse Cardiomyocytes (NMCMs)

Primary NMCMs were isolated from 1‐ to 3‐day‐old neonatal mice under sterile conditions. Briefly, the hearts were harvested, rinsed three times with pre‐cooled sterile PBS (Solarbio, P1010) containing 1% penicillin–streptomycin to remove residual blood, and minced into approximately 1 mm^3^ fragments. The tissues were subjected to overnight digestion with 0.25% trypsin at 4°C for 12–16 h. Subsequently, Type II collagenase (MCE, HY‐E70005B; final concentration 0.8 mg/mL) was added, and the suspension was incubated in a 37°C water bath for 15 min to complete digestion. The process was terminated by adding DMEM supplemented with 10% fetal bovine serum (FBS). After centrifugation at 200 × *g* for 5 min, the cell pellet was resuspended in DMEM containing 15% FBS and 1% penicillin–streptomycin. To purify the cardiomyocytes, the cell suspension was subjected to differential pre‐plating for 2 h at 37°C in a 5% CO_2_ incubator, allowing fibroblasts to preferentially adhere to the dish. The supernatant containing NMCMs was then collected and seeded into new culture dishes. The culture medium was replaced 24 h post‐seeding to remove non‐adherent cells. After 48 h of culture, the viability and purity of NMCMs were confirmed by observing their characteristic polygonal morphology and spontaneous rhythmic contractions under an inverted phase‐contrast microscope.

#### 4.2.2 Transfection of Primary Cardiomyocytes

When the confluency of primary cardiomyocytes reached 70%–80%, transfection was performed using Lipofectamine RNAiMAX transfection reagent (Invitrogen, 13778075). siRNA and RNAiMAX were separately diluted with Opti‐MEM medium (the final concentration of siRNA was adjusted to 50 nM, and the volume ratio of RNAiMAX to siRNA was 1.5:1). After being incubated at room temperature for 5 min, the two diluted solutions were mixed, gently pipetted to ensure homogeneity, and then incubated at room temperature for 20 min to form transfection complexes. The transfection complexes were evenly added dropwise to the cell culture dish, and the dish was gently shaken to distribute the complexes uniformly. The dish was then placed in a 37°C, 5% CO_2_ incubator for culture. 6 h after transfection, the medium was replaced with complete DMEM medium containing 15% fetal bovine serum and 1% penicillin–streptomycin. The cells were further cultured for 48 h, after which they were harvested for subsequent detection.

### 4.3 Western Blotting

#### 4.3.1 Protein Extraction

Total protein was extracted from mouse heart tissues or primary cardiomyocytes using RIPA lysis buffer supplemented with 1% protease and phosphatase inhibitor cocktails (Beyotime, ST507). Heart tissues were homogenized thoroughly on ice. The lysates were incubated on ice for 30 min and subsequently centrifuged at 12,000 × *g* for 15 min at 4°C. Protein concentration was determined using a BCA Protein Assay Kit (Aikerui Bio, AG11728) to ensure equal loading.

#### 4.3.2 Electrophoresis and Transfer

Protein samples (50 μg/lane) were mixed with 5× SDS loading buffer and denatured at 98°C for 5 min. The proteins were separated by 10% SDS‐PAGE and then transferred onto PVDF membranes (Millipore, IPFL00010) via a wet transfer system (300 mA, 90 min). Membranes were blocked with 5% non‐fat milk (Solarbio, D8430) in TBST for 1 h at room temperature.

#### 4.3.3 Immunoblotting and Visualization

The membranes were incubated overnight at 4°C with the following primary antibodies: GAPDH antibody (Proteintech, 60004–1‐Ig), Lamin A/C antibody (Santa Cruz, SC‐376248), Lamin A antibody (Abcam, ab26300), METTL14 antibody (Sigma‐Aldrich, HPA038002), METTL3 antibody (Abcam, ab240595), P16 antibody (Abcam, ab54210), P21 antibody (Abcam, ab227443), IL6 antibody (CST, 12912T), and Alpha Actinin antibody (Abcam, ab137346). After washing three times with TBST, the membranes were incubated with HRP‐conjugated secondary antibodies (1:5000, Abcam, ab6721) for 1 h at room temperature. Protein bands were visualized using an ECL Chemiluminescence Kit (Millipore, WBKLS0500) and quantified by densitometry using ImageJ software. Target protein levels were normalized to GAPDH as an internal control.

### 4.4 Methylated RNA Immunoprecipitation (MeRIP)

#### 4.4.1 Tissue Lysis and Pre‐Treatment

Mouse heart tissues were rinsed with pre‐cooled PBS and thoroughly homogenized in RIP lysis buffer supplemented with a protease inhibitor cocktail and RNase inhibitors. The homogenate was centrifuged at 1500 × *g* for 5 min to collect the cell pellet. The pellet was then resuspended in pre‐cooled RIP lysis buffer and lysed on ice for 30 min with intermittent pipetting. Following centrifugation at 12,000 × *g* for 15 min at 4°C, the supernatant was collected. A portion of the supernatant was reserved as Input, while the remainder was used for immunoprecipitation.

#### 4.4.2 Immunoprecipitation (IP)

Magnetic beads were pre‐conjugated with an m^6^A‐specific antibody or an anti‐IgG control antibody by overnight incubation at 4°C. The bead‐antibody complexes were washed three times with RIP washing buffer. Subsequently, 100 μL of the protein‐RNA lysate was added to the complexes and incubated overnight at 4°C with gentle rotation. The following day, the beads were washed five times with RIP washing buffer to remove non‐specifically bound RNA.

#### 4.4.3 RNA Recovery and Reverse Transcription

To dissociate the protein‐RNA complexes, the beads were incubated with Proteinase K at 55°C for 30 min. RNA was then extracted using the phenol‐chloroform method, followed by ethanol precipitation. The purity (A260/A280 ratio: 1.8–2.0) and concentration of the RNA samples were verified using a NanoDrop 2000 spectrophotometer. cDNA was synthesized using the PrimeScript RT Reagent Kit (TaKaRa, RR037A) according to the manufacturer's instructions.

#### 4.4.4 qRT‐PCR and Statistical Analysis

Quantitative real‐time PCR (qRT‐PCR) was performed on a qTOWER3 system using Hieff qPCR SYBR Green Master Mix (Yeasen Biotech, 11201ES03). Relative enrichment was calculated using the 2^−ΔΔCt^ method. Statistical analysis was conducted using GraphPad Prism 9.0. Data are presented as mean ± s.e.m. Significant differences between groups were determined using an unpaired *t*‐test, with *p* < 0.05 considered statistically significant.

### 4.5 Calcium Transient Imaging

Primary neonatal mouse ventricular cardiomyocytes seeded on glass‐bottom coverslips were loaded with 5 μM Fluo‐4 AM (Thermo Fisher Scientific, F14201) supplemented with 0.05% (*w*/*v*) Pluronic F‐127 for 20 min at 37°C in the dark for calcium indicator loading. Following loading, cells were washed twice with pre‐warmed modified Tyrode's solution (140 mM NaCl, 5.4 mM KCl, 1.8 mM CaCl_2_, 1 mM MgCl_2_, 5 mM HEPES, 5.5 mM glucose; pH 7.4), followed by an additional 20 min incubation at room temperature to permit complete intracellular de‐esterification of AM groups. Time‐series recordings were acquired using a Zeiss confocal microscope (Zen 3.3 software).

### 4.6 mRNA Stability Assay

Primary neonatal mouse cardiomyocytes were treated with 5 μg/mL actinomycin D (ActD; MCE, HY‐17559) to block de‐novo transcription. Total RNA was harvested at sequential time points following ActD treatment. RNA was isolated and reverse‐transcribed, and transcript abundance of target genes was quantified by qRT‐PCR. The relative remaining mRNA levels at each time point were normalized to the 0 h time‐point. mRNA half‐life was calculated by fitting the decay curve to a one‐phase exponential decay model using GraphPad Prism9.0.

### 4.7 Nanoindentation Test

The nanomechanical properties of myocardial tissues were measured using a nanoindenter calibrated systematically with fused silica reference samples to ensure the accuracy of the indenter area function and system stiffness. Indentation parameters were set at a loading/unloading rate of 0.08 mN/s with an 8 s dwell time. Frozen cardiac sections were securely fixed onto the sample stage, and intact left ventricular myocardial regions without visible cracks were localized as target areas using an integrated optical microscope. For each sample, five non‐overlapping regions of interest (ROIs) were randomly selected, with six independent indentation sites performed in each ROI; a minimum spacing of 50 μm was maintained between adjacent indentations to prevent mechanical crosstalk. Experimental data were processed using Bruker Nano Suite software to calculate the elastic modulus and hardness based on the Oliver‐Pharr model. Statistical analysis was performed with GraphPad Prism 9.0, where data were expressed as mean ± s.e.m., and intergroup differences were assessed using the unpaired *t*‐test with *p* < 0.05 considered statistically significant.

### 4.8 Masson Staining

Mouse heart tissues were fixed in 4% paraformaldehyde (Sangon Biotech) for 24 h, followed by dehydration through a graded series of ethanol, clearing in xylene, and embedding in paraffin. Sections (5 μm thick) were prepared and subsequently deparaffinized and rehydrated for histological staining. Masson's trichrome staining was performed using a Modified Masson Trichrome Staining Kit (Solarbio, G1346) according to the manufacturer's instructions. Briefly, sections were stained with hematoxylin for 5 min, differentiated with 1% hydrochloric acid alcohol for 30 s, and then stained for muscle and collagen using Eosin, phosphomolybdic acid, and aniline blue sequentially. After staining, sections were dehydrated, cleared with xylene, and mounted with neutral balsam. Representative images were captured using an optical microscope (Olympus, BX53).

### 4.9 Immunofluorescence Staining

Mouse heart tissues were embedded in OCT compound (Sakura, 4583) and cryosectioned into 8 μm‐thick slices at −20°C. The sections were washed thrice with PBS (5 min each), permeabilized with 0.3% Triton X‐100 (Sigma) for 15 min, and blocked with 5% BSA (Solarbio) at room temperature for 1 h. Subsequently, the sections were incubated overnight at 4°C with primary antibody. After washing three times with PBS, the sections were incubated with a FITC‐conjugated secondary antibody (1:500, Abcam, ab6717) for 1 h at room temperature in the dark. Nuclei were counterstained with DAPI (Solarbio) for 5 min. Finally, the sections were mounted with antifade mounting medium (Solarbio) and visualized under a fluorescence microscope.

### 4.10 Statistical Analysis

All experimental data were statistically analyzed using GraphPad Prism 9.0 software. Data are presented as mean ± s.e.m. The independent samples *t*‐test was used for comparisons between two groups. The Log‐rank test was used for survival analysis. *p*‐value < 0.05 was considered statistically significant, *p < 0.05, **p < 0.01, ***p < 0.001. Post hoc power analysis using G*Power 3.1 confirmed that the primary endpoints were adequately powered (power ≥ 80%). Some secondary endpoints were exploratory and should be interpreted accordingly.

## Author Contributions

S.W. and J.Z. conceived and performed the core experiments, analyzed the data, and co‐wrote the initial draft. Y.A. and D.R. participated in partial in vitro and animal experiments, assisting in verification of the key results. C.Y., C.X., M.W., M.L., X.H., J.Z., R.M., Z.Z., J.S., J.D., Y.S., and Z.W. contributed to sample collection, processing, preservation, and partial data analysis, providing essential research support. As the corresponding authors, J.Z., W.S., and B.L. supervised the entire project, designed the study, optimized protocols, resolved technical issues, and revised the manuscript.

## Funding

This work was supported by grants from the National Natural Science Foundation of China (82488301, 82125012, 82401821, and 32430048), the Shenzhen Medical Research Fund (C2406001), the National Key R&D Program of China (2024YFA0918700, 2023YFC2509900, and 2025YFC3608100), the Shenzhen Science and Technology Talent Project (RCYX20231211090127031), the Shenzhen Key Laboratory of Cell Senescence and Regeneration Innovation (2024548181), the Shenzhen University 2035 Program for Excellent Research (0000214), the Shenzhen Municipal Commission of Science and Technology Innovation (JCYJ20220818100016035 and JCYJ20220818100009020), and China Postdoctoral Science Foundation (Grant 2024M762133). The funders had no role in the preparation of the manuscript or the decision to publish it.

## Conflicts of Interest

The authors declare no conflicts of interest.

## Supporting information


**Figure S1:** DCM‐associated genes and progerin induction in *Myh6‐*Cre; *Lmna* ^fl/fl^ primary mouse cardiomyocytes.

**Figure S2:** Cardiac molecular and functional phenotypes of *Lmna* ^G609G/G609G^ and *Myh6‐*Cre; *Lmna* ^fl/fl^ mice.

**Figure S3:** Altered METTL3/METTL14 expression in aging and *Lmna* knockout mouse tissues.

**Figure S4:** METTL14 expression upon ISO stimulation, AAV‐cTNT‐METTL14‐GFP vector construction, and cardiac senescence gene profiling in *Zmpste24* ^−/−^ mice.

**Table S1:** Primer sequences and statistical summary.

## Data Availability

All the generated data and the analysis developed in this study are included in this article.
