# Supplementary material for: METTL14‐Mediated m6A Methylation Preserves Cardiomyocyte Homeostasis and Attenuates Progeria‐Associated Cardiac Remodeling
Source: Aging Cell. 2026 Oct 15;25(10):e70765. doi: 10.1111/acel.70765 (PMC13670420; doi:10.1111/acel.70765)

A

Dilated Cardiomyopathy - DCM

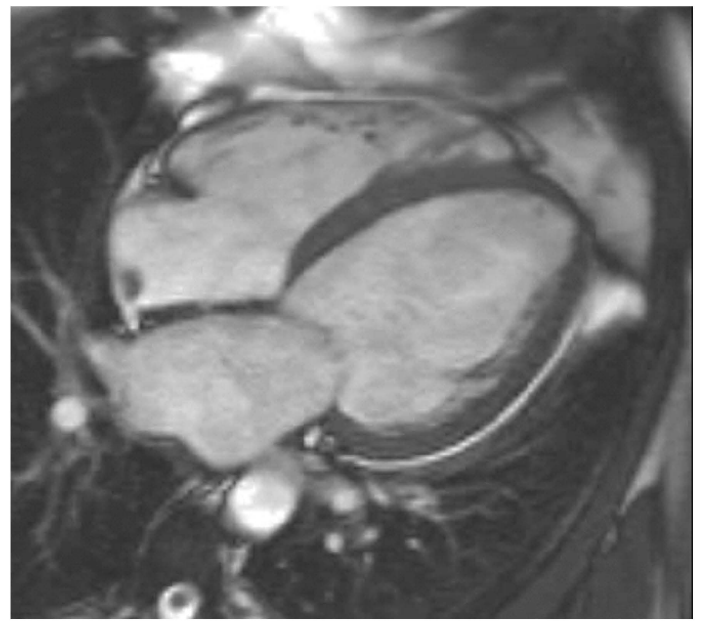

*TTN - DSP - MYH7 - **LMNA** - TNNT2 - TPM1 VCL-TCAP - LDB3 - MYBPC3 - ABCC9 - DES TNNI3 - ACTC1 - SGCD - PLN - TAZ*

Excess of *LMNA* variants in DCM

	All Vars	Truncating	Non-Truncating
Excess in DCM	0.0378	0.0152	0.0227
	$p<0.0001$	$p<0.0001$	$p<0.0001$
Etiological fraction	0.86	0.99	0.79
	0.81 - 0.90	0.98 - 1.00	0.69 - 0.86
Odds Ratio	7.35	99.61	4.84
	5.24 - 10.11	40.10 - 269.28	3.20 - 7.10

B

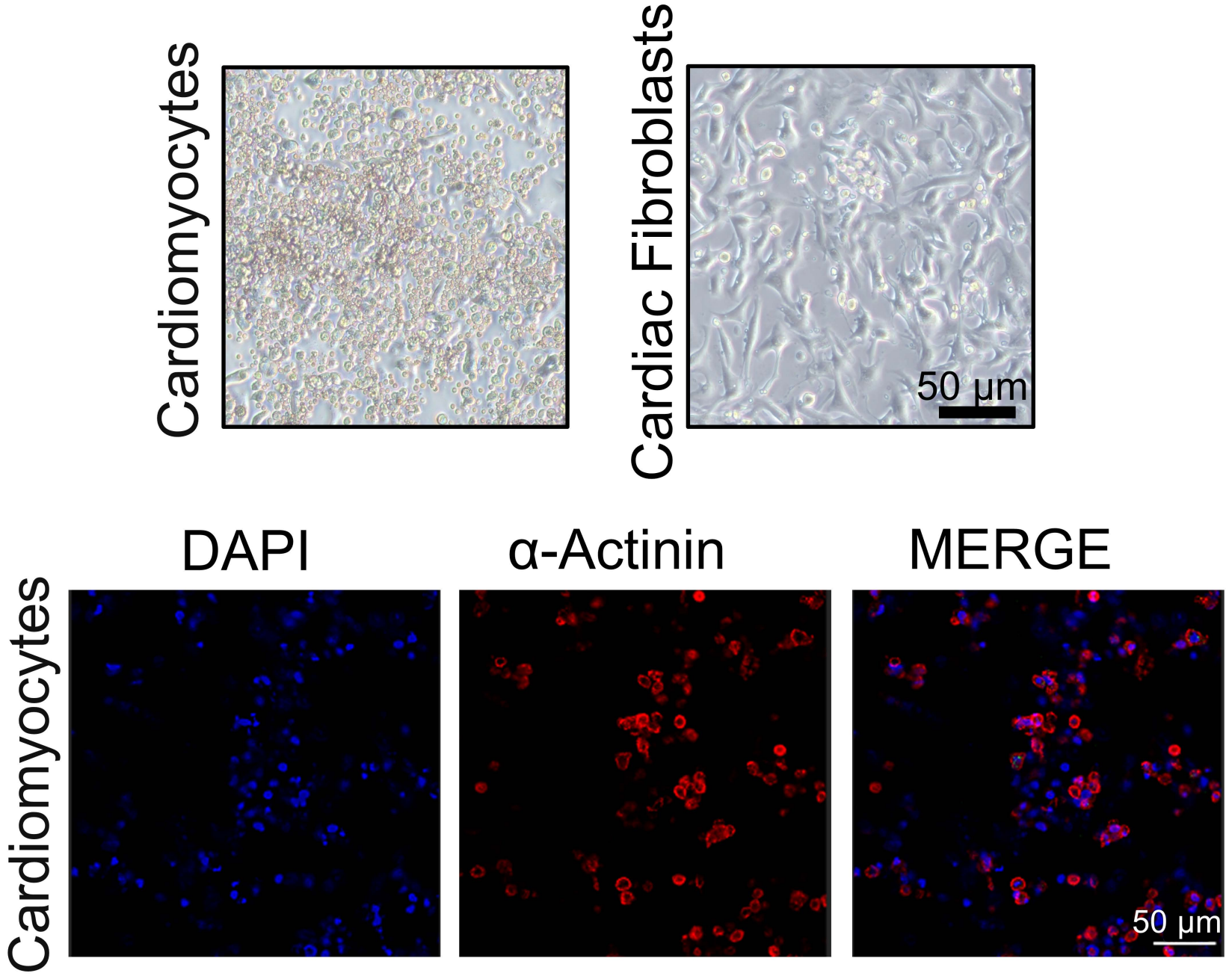

C

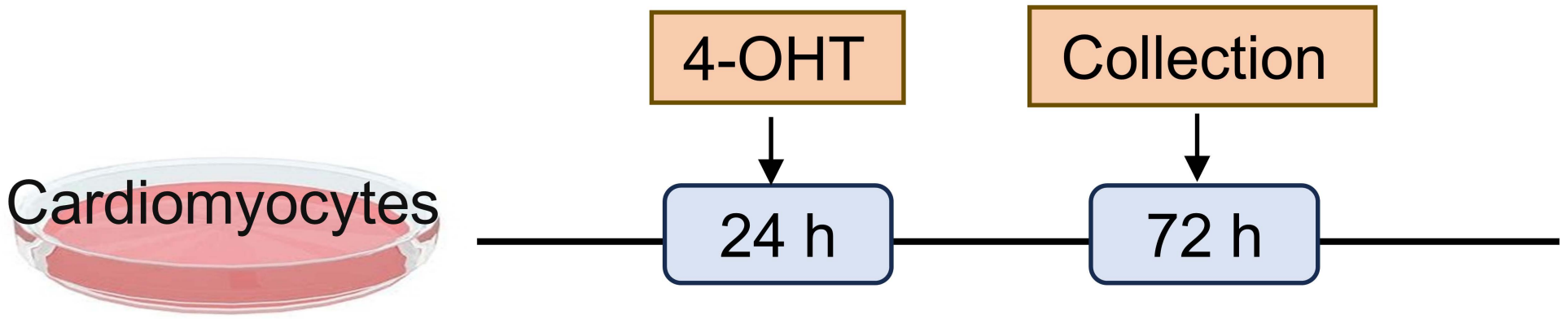

D

Mouse Primary Cardiomyocytes

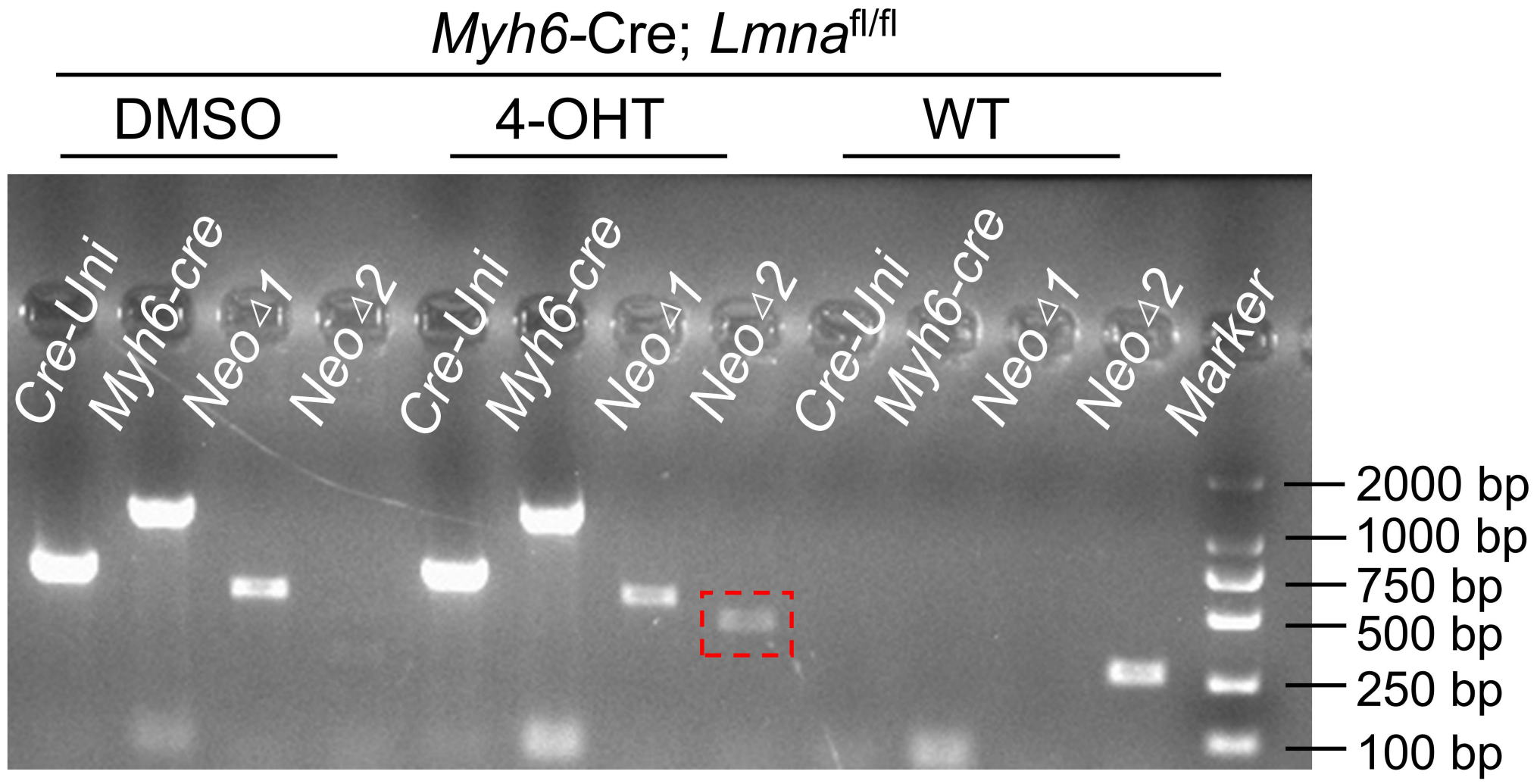

Supplement: Supplementary file 1 [file ACEL-25-e70765-s001.pdf]
