# Supplementary material for: METTL14‐Mediated m6A Methylation Preserves Cardiomyocyte Homeostasis and Attenuates Progeria‐Associated Cardiac Remodeling
Source: Aging Cell. 2026 Oct 15;25(10):e70765. doi: 10.1111/acel.70765 (PMC13670420; doi:10.1111/acel.70765)
Supplement: Supplementary file 2 [file ACEL-25-e70765-s002.pdf]

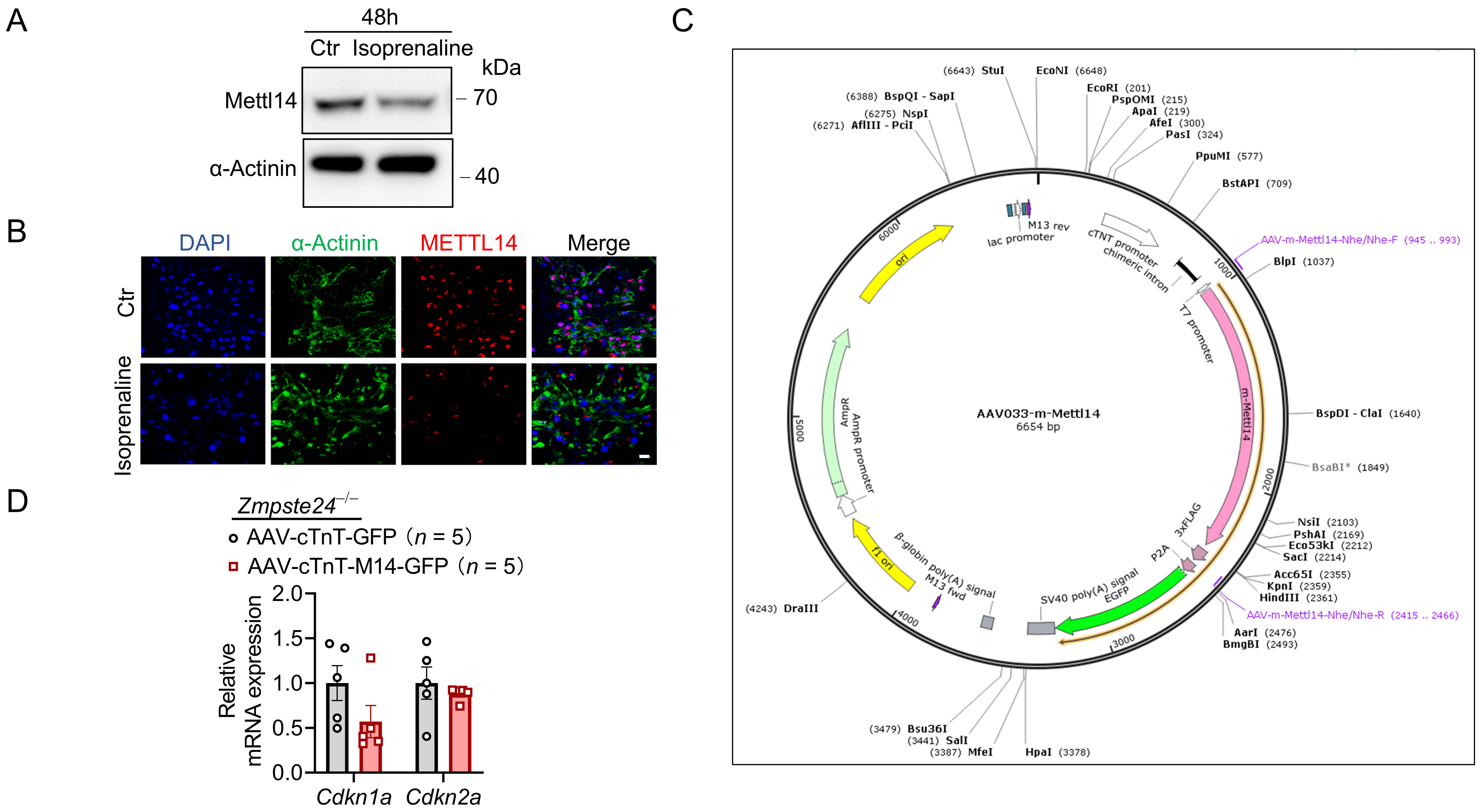
A
48h
Ctr
Isoprenaline
kDa
Mettl14
70
α-Actinin
40
B
DAPI
α-Actinin
METTL14
Merge
Ctr
Isoprenaline
C
AAV033-m-Mettl14
6654 bp
(6643) StuI
EcoNI (6648)
EcoRI (201)
PspOMI (215)
ApaI (219)
AfeI (300)
PasI (324)
PpuMI (577)
BstAPI (709)
(6388) BspQI - SapI
(6275) NspI
(6271) AflIII - PciI
AAV-m-Mettl14-Nhe/Nhe-F (945 .. 993)
BlpI (1037)
BspDI - ClaI (1640)
BsaBI* (1849)
NsiI (2103)
PshAI (2169)
Eco53kI (2212)
SacI (2214)
Acc65I (2355)
KpnI (2359)
HindIII (2361)
AAV-m-Mettl14-Nhe/Nhe-R (2415 .. 2466)
AarI (2476)
BmgBI (2493)
(4243) DraIII
(3479) Bsu36I
(3441) SalI
(3387) MfeI
HpaI (3378)
M13 rev
lac promoter
cTNT promoter
chimeric intron
T7 promoter
m-Mettl14
3xFLAG
P2A
EGFP
SV40 poly(A) signal
β-globin poly(A) signal
M13 fwd
f1 ori
AmpR
AmpR promoter
ori
D
Zmpste24−/−
AAV-cTnT-GFP (n = 5)
AAV-cTnT-M14-GFP (n = 5)
Relative mRNA expression
2.0
1.5
1.0
0.5
0.0
Cdkn1a
Cdkn2a
