# Supplementary material for: METTL14‐Mediated m6A Methylation Preserves Cardiomyocyte Homeostasis and Attenuates Progeria‐Associated Cardiac Remodeling
Source: Aging Cell. 2026 Oct 15;25(10):e70765. doi: 10.1111/acel.70765 (PMC13670420; doi:10.1111/acel.70765)
Supplement: Supplementary file 3 [file ACEL-25-e70765-s003.pdf]

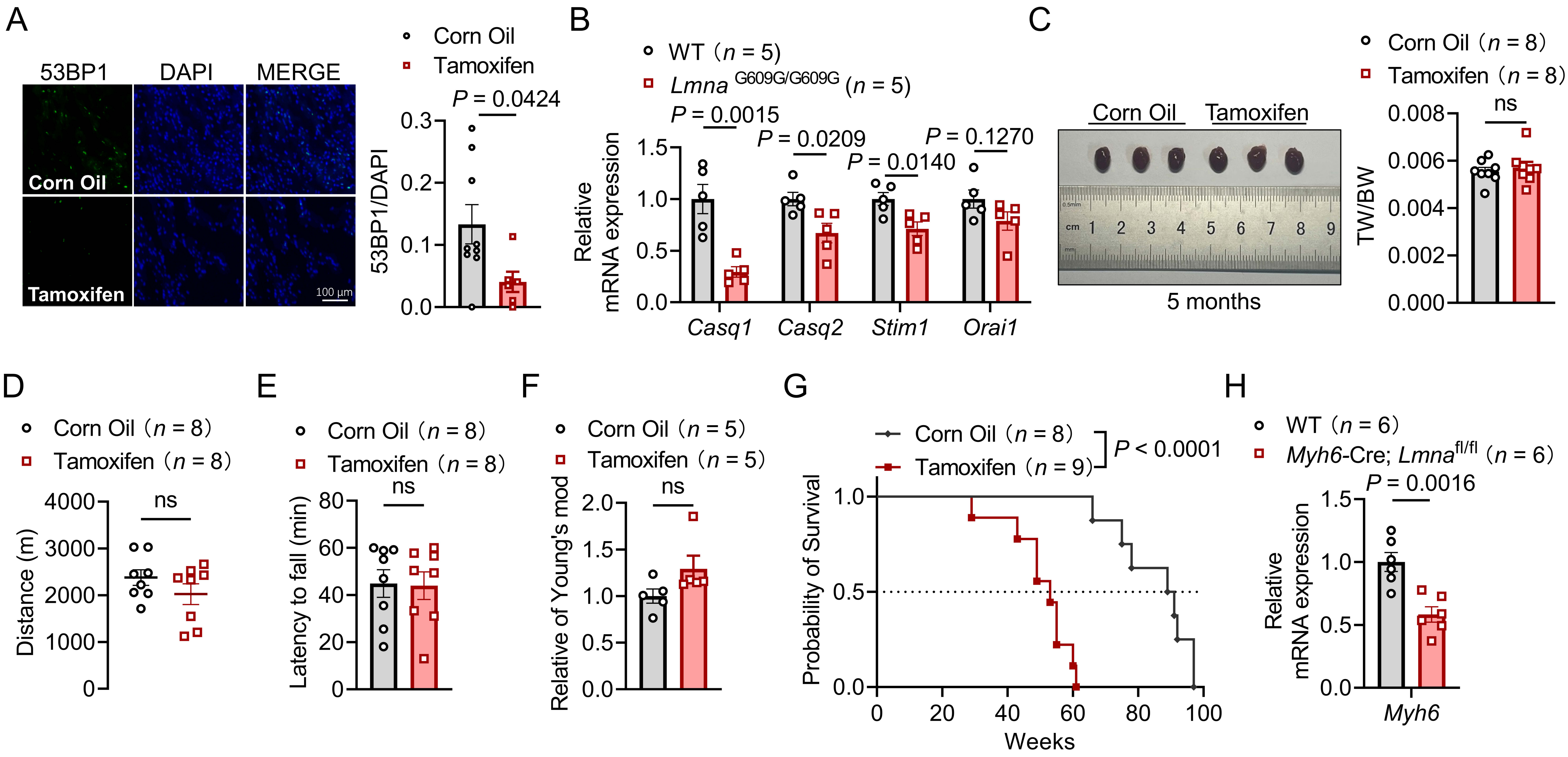

A
53BP1
DAPI
MERGE
Corn Oil
Tamoxifen
100 μm
Corn Oil
Tamoxifen
P = 0.0424
53BP1/DAPI
B
WT (n = 5)
Lmna G609G/G609G (n = 5)
P = 0.0015
P = 0.0209
P = 0.0140
P = 0.1270
Relative mRNA expression
Casq1
Casq2
Stim1
Orai1
C
Corn Oil
Tamoxifen
5 months
Corn Oil (n = 8)
Tamoxifen (n = 8)
ns
TW/BW
D
Corn Oil (n = 8)
Tamoxifen (n = 8)
ns
Distance (m)
E
Corn Oil (n = 8)
Tamoxifen (n = 8)
ns
Latency to fall (min)
F
Corn Oil (n = 5)
Tamoxifen (n = 5)
ns
Relative of Young's mod
G
Corn Oil (n = 8)
Tamoxifen (n = 9)
P < 0.0001
Probability of Survival
Weeks
H
WT (n = 6)
Myh6-Cre; Lmna fl/fl (n = 6)
P = 0.0016
Relative mRNA expression
Myh6
