# Supplementary material for: METTL14‐Mediated m6A Methylation Preserves Cardiomyocyte Homeostasis and Attenuates Progeria‐Associated Cardiac Remodeling
Source: Aging Cell. 2026 Oct 15;25(10):e70765. doi: 10.1111/acel.70765 (PMC13670420; doi:10.1111/acel.70765)
Supplement: Supplementary file 4 [file ACEL-25-e70765-s004.pdf]

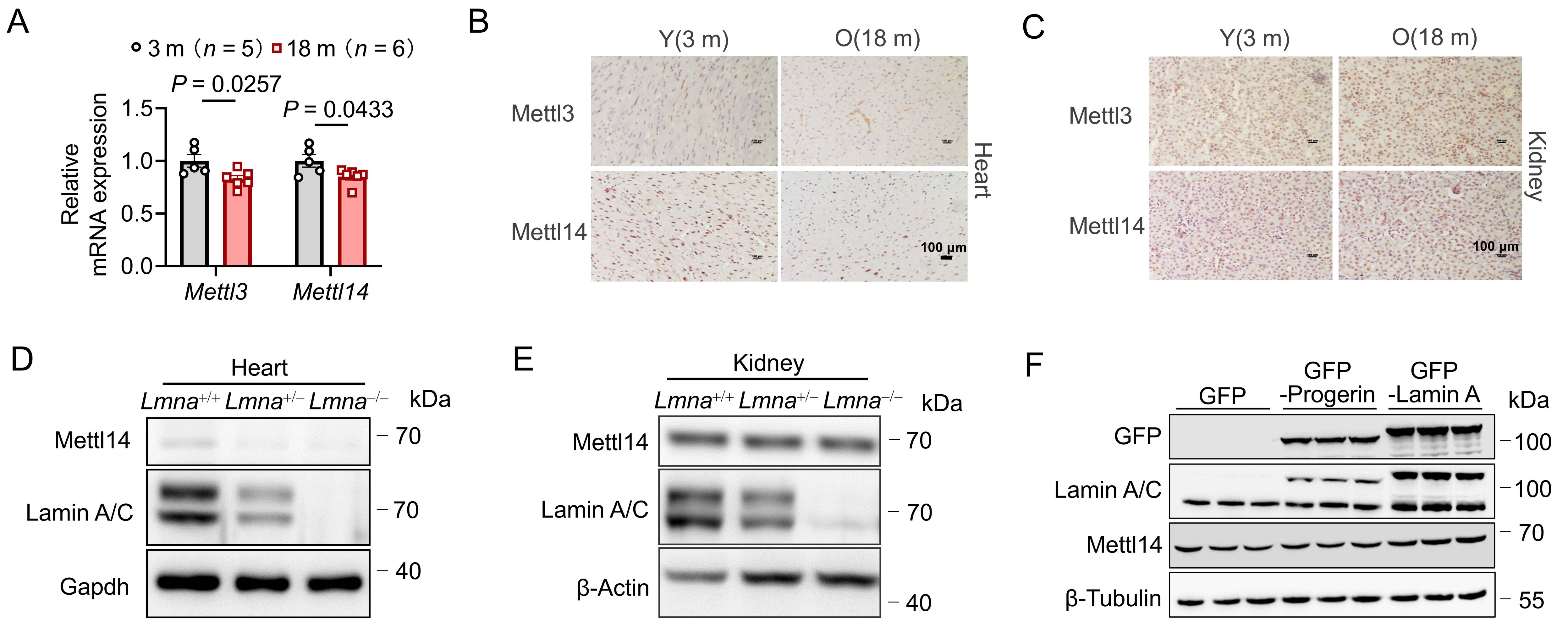

A
3 m (n = 5) 18 m (n = 6)
P = 0.0257
P = 0.0433
Relative mRNA expression
1.5
1.0
0.5
0.0
Mettl3
Mettl14
B
Y(3 m)
O(18 m)
Mettl3
Mettl14
Heart
100 μm
C
Y(3 m)
O(18 m)
Mettl3
Mettl14
Kidney
100 μm
D
Heart
Lmna+/+ Lmna+/− Lmna−/−
kDa
Mettl14
Lamin A/C
Gapdh
70
70
40
E
Kidney
Lmna+/+ Lmna+/− Lmna−/−
kDa
Mettl14
Lamin A/C
β-Actin
70
70
40
F
GFP
GFP -Progerin
GFP -Lamin A
kDa
GFP
Lamin A/C
Mettl14
β-Tubulin
100
100
70
55
